# Endoscopic-assisted lateral neck dissection and open lateral neck dissection in the treatment of lateral neck lymph node metastasis in papillary thyroid carcinoma: A comparison of therapeutic effect

**DOI:** 10.12669/pjms.38.7.5826

**Published:** 2022

**Authors:** Tao Ma, Shuai Zhang, Dongmei Huang, Gang Zhang, Boyi Chen, Ning Zhang

**Affiliations:** 1 Tao Ma, Department of General Surgery, Baoding No.1 Central Hospital, Baoding, 071000, Hebei, China; 2 Shuai Zhang, Department of General Surgery, Baoding No.1 Central Hospital, Baoding, 071000, Hebei, China; 3 Dongmei Huang, Department of Thyroid and Neck Tumor, Tianjin Medical University Cancer Institute and Hospital, National Clinical Research Center for Cancer, Key Laboratory of Cancer Prevention and Therapy, Tianjin’s Clinical Research Center for Cancer, Tianjin 300060, China; 4 Gang Zhang, Department of General Surgery, Baoding No.1 Central Hospital, Baoding, 071000, Hebei, China; 5 Boyi Chen, Department of General Surgery, Baoding No.1 Central Hospital, Baoding, 071000, Hebei, China; 6 Ning Zhang, Department of General Surgery, Baoding No.1 Central Hospital, Baoding, 071000, Hebei, China

**Keywords:** Endoscopic-assisted, Open lateral neck dissection, Papillary thyroid carcinoma, Lymph node metastasis

## Abstract

**Objectives:**

To compare the therapeutic effect of endoscopic-assisted lateral neck dissection and open lateral neck dissection in the treatment of lateral neck lymph node metastasis of patients with papillary thyroid carcinoma (PTC).

**Methods:**

A retrospective analysis was carried out focusing on the general clinical data of 86 patients with PTC treated Baoding No.1 Central Hospital from January 2020 to September 2021. According to different surgical methods, enrolled patients were divided into the endoscopic surgery group (n = 34) and the open surgery group (n = 52). Further comparison was performed on the operation indexes [operation time, postoperative length of stay in hospital, number of dissected lymph nodes (central area, lateral cervical area), number of metastatic lymph nodes (central area, lateral cervical area), hospitalization cost], postoperative complications, postoperative neck pain, neck numbness discomfort score, and satisfaction with postoperative cosmetic effect.

**Results:**

The operation time and hospitalization cost of the endoscopic surgery group were higher than those of the open surgery group, and the intraoperative blood loss was lower than that of the open surgery group, with statistically significant differences (*p<*0.05). There was no significant difference in the length of stay in the hospital, the number of dissected lymph nodes, the number of metastatic lymph nodes and the detection rate of lymph nodes in zone II between the two groups (*p>*0.05). Furthermore, a statistically significant difference was observed in the incidence of postoperative complications between the two groups, which was lower in the endoscopic surgery group (29.4%) than that in the open surgery group (51.9%) (*p<*0.05). There was no significant difference in postoperative neck pain scores between the two groups (*p>*0.05). While the postoperative neck numbness discomfort score, and satisfaction score with postoperative cosmetic effect in the endoscopic surgery group were better than those in the open surgery group, and the difference was statistically significant (*p<*0.05).

**Conclusion:**

Endoscopic-assisted lateral neck dissection can reduce intraoperative blood loss and postoperative complication incidence in the treatment of lateral neck lymph node metastasis of PTC. However, it has the disadvantages of longer operation time and high hospitalization costs.

## INTRODUCTION

Thyroid cancer is the most common malignant tumor of the endocrine system, and its incidence in females is significantly higher than that in males.^1^ Papillary thyroid carcinoma (PTC) comprises almost 80% of all thyroid cancers.^2^ Traditional open surgery is the gold standard for the treatment of PTC, and its 10-year survival rate reaches 90% after treatment.^3^ However, during lymph node dissection in the lateral neck in the process of traditional open surgery, it is necessary to make “L”-shaped, reverse “L”- or long “collar”-shaped incision in the patient’s neck.^4^ It may produce neck scars that cannot be covered, which will have an adverse physical and psychological impact on the patients. With the development of the concept of minimally invasive surgery, endoscopy in surgery is becoming more widely available in the clinical setting. Endoscopic thyroid surgery includes total endoscopic thyroid surgery and endoscopic-assisted thyroid surgery. Among them, endoscopic-assisted thyroid surgery can expose the anatomical structure of cervical nerves and blood vessels, and has the advantages of simple operation, beginner-friendly, small separation wound, small incision and short learning curve, which has a wider application in clinical practice. The safety and cosmetic effect are the primary concerns of current clinical research on endoscopic and open surgeries in the treatment of PTC.^5^ The present research was a retrospective study to analyze the surgical indexes, postoperative complications, postoperative neck pain, neck numbness discomfort score, and the satisfaction of postoperative cosmetic effect. It is expected to better understand the advantages and disadvantages of both surgeries and provide a potential reference for clinical practice.

## METHODS

A retrospective analysis was carried out focusing on the general clinical data of 86 patients with PTC treated in Baoding No.1 Central Hospital from January 2020 to September 2021. According to different surgical methods, enrolled patients were divided into endoscopic surgery group (n = 34) and open surgery group (n = 52). In the endoscopic surgery group, there were three males and 31 females, with an average age of (31.4±7.4) years old (19~58 years); The type of operation was total thyroidectomy in 12 cases and unilateral lobectomy in 22 cases, including five cases on the left, 10 cases on the right and 19 cases on both sides. While in the open surgery group, there were 10 males and 42 females, with an average age of (33.9±7.3) years old (20~58 years); The operation type was total thyroidectomy in 17 cases and unilateral lobectomy in 35 cases, including 13 cases on the left, 23 cases on the right and 16 cases on both sides

### Ethical Approval

This study was approved by the Institutional Ethics Committee of Baoding No.1 Central Hospital on September 20, 2022 (No.2022033), and written informed consent was obtained from all participants.

### Inclusion criteria:

- Patients who met the diagnostic criteria of PTC in the Guidelines of the American Thyroid Association (ATA)^6^;
- The newly treated patients who had no previous history of neck radiotherapy
- Patients with tumor diameter ≤3cm and who had received lateral neck lymph node dissection within the selective period of stay in the hospital^7^;
- Patients with the maximum diameter of metastatic lymph nodes ≤2cm.

### Exclusion criteria

- Patients with cancer cells spreading distally;
- Patients with other malignant tumors;
- Patients who were converted from endoscopic surgery to open surgery;
- Patients with lymph node fusion and tumor invasion of trachea, esophagus, nerve, etc.^8^

There was no significant difference in general data (gender, age, type of operation, dissection side) between the two groups (*p>*0.05). This study was approved by the Medical Ethics Committee of our hospital, and all patients had provided informed consent.

Open lateral neck dissection was performed in the open surgery group: After routine anesthesia, an arc-shaped incision of about 12 ~ 15 cm in length was made along the dermatoglyph at 1.5 ~ 2.0 cm above the anterior cervical sternal notch. The location of the incision was close to the side of the dissection. The skin and platysma were cut layer by layer, which was up to the level of the digastric muscle, down to the clavicle, and outside to the posterior edge of the sternocleidomastoid muscle. Pay attention to avoid cervical veins and nerve plexus. Patients in this group were provided with total thyroidectomy + central lymph node dissection or hemithyroidectomy and isthmectomy + central lymph node dissection on the affected side. Intraoperatively, the accessory nerve was dissociated and protected, the sternocleidomastoid muscle was dissociated, associated with the dissociation of lymph nodes and soft tissues in the lateral area of the internal jugular vein; meanwhile, attention was paid to expose and protect the greater auricular nerve and the lesser occipital nerve, cut and ligate the transverse carotid artery and vein, and sharply separate the internal jugular vein from the surface of the internal jugular vein; the enlarged lymph nodes and soft tissues in zones II, III and IV were completely removed along the surface of deep cervical fascia; then, the venous angle was sutured carefully, followed by the dissociation of the soft tissue inside the internal jugular vein, exposure of the carotid sheath, exposure and protection of the internal jugular vein and vagus nerve, and removal the lymph nodes and soft tissue inside the internal jugular vein. Postoperatively, additional attention was attached to the examination of bleeding points, lymphatic leakage, as well as the integrity of nerves and vessels.^9^ The incision was sutured after catheterization for drainage and hemostasis.

Endoscopic assisted lateral neck dissection was adopted in the endoscopic surgery group:^10^ The incision was made at 1.0 ~ 1.5 cm above the anterior cervical sternal notch, with a length of about 6cm. The thyroid and central lymph nodes were treated like that in the open surgery group. Endoscopic instruments were placed into the anterior cervical incision. The layers of incision were the same as those in the open surgery group, followed by the construction of the operation space and determination of the range of operation. Intraoperatively, the accessory nerve was dissociated and protected by using an ultrasonic knife, the sternocleidomastoid muscle was dissociated, and the lymph nodes and soft tissues were also dissociated in the lateral area of the internal jugular vein; a further operation was performed to expose and protect the greater auricular nerve and the lesser occipital nerve, cut and ligate the transverse carotid artery and vein, and separate the internal jugular vein; the enlarged lymph nodes and soft tissues in zones II, III and IV were completely removed along the surface of deep cervical fascia; and the venous angle was sutured carefully, followed by the dissociation of the soft tissue inside the internal jugular vein, exposure of the carotid sheath, exposure and protection of the internal jugular vein and vagus nerve, and removal the lymph nodes and soft tissue inside the internal jugular vein. Postoperatively, with the bleeding point, lymphatic leakage, the integrity of nerves and vessels confirmed, the incision was sutured after catheterization for drainage and hemostasis.

### Surgical indexes

Operation time, postoperative length of stay in the hospital, number of dissected lymph nodes (central area, lateral cervical area), number of metastatic lymph nodes (central area, lateral cervical area), and hospitalization cost.

### Postoperative complications^11^

Hoarseness, hypoparathyroidism, hypocalcemia and postoperative hematoma.

Postoperative neck pain, neck numbness discomfort score, and satisfaction with a postoperative cosmetic effect: All scores were evaluated by subjective evaluation method. Three days after the operation, according to the presence of pain, the scores of pain were: one point, no pain; two points, mild pain; three points, moderate pain; four points, severe pain; and five points, extremely severe pain.^12^ Neck numbness discomfort was evaluated one month after operation (one point: no discomfort, two points: mild discomfort, three points: moderate discomfort, four points: severe discomfort and five points: extremely severe discomfort).^13^ In addition, at three months after the operation, the cosmetic effect satisfaction score was given, including one point of very satisfied, two points of satisfied, three points of generally satisfied, 4 points of dissatisfied and five points of very dissatisfied.^14^

### Statistical Analysis

SPSS 26.0 statistical software was used for statistical analysis. The measurement data conforming to the normal distribution were expressed by mean±standard deviation (Mean±SD), and the t-test was used for the comparison between groups. While the measurement data that did not conform to the normal distribution were expressed by median and interquartile spacing, and comparison between groups was realized by using the Mann-Whitney U test. The counting data were expressed by rate and compared between groups using the χ^2^ test. P<0.05 meant that the difference was statistically significant.

## RESULTS

As shown in Table-I, the operation time and hospitalization cost of the endoscopic surgery group were higher than those of the open surgery group, and the intraoperative blood loss was lower than that of the open surgery group, with statistically significant differences (*p<*0.05). Table-I. There was no significant difference in the length of stay in the hospital, the number of dissected lymph nodes, the number of metastatic lymph nodes and the detection rate of lymph nodes in zone II between the two groups (*p>*0.05).

**Table I:** Comparison of surgical indexes between the two groups.

	Endoscopic surgery group (n=34)	Open surgery group (n=52)	t/Z	p
Operation time (min)	172.6±44.0	137.2±31.9	4.329	0.001
Blood loss (ml)	47.7±10.1	60.5±13.4	4.775	0.001
Postoperative length of stay in hospital (d)	6.0(4.0, 7.0)	6.0(5.0, 6.8)	0.673	0.501
Number of dissected lymph nodes (n)				
Central area	10.5(8.0, 15.0)	9.0(5.0, 15.0)	1.762	0.078
Lateral cervical area	27.0(22.0, 36.5)	27.5(18.3, 37.5)	0.588	0.557
Number of metastatic lymph nodes (n)				
Central area	4.0(1.8, 7.3)	3.5(2.0, 6.0)	0.382	0.703
Lateral cervical area	4.0(1.0, 6.0)	3.0(1.0, 4.0)	1.033	0.302
Number of lymph nodes in zone II (n)	10.0(5.8, 15.5)	7.0(4.0, 11.8)	1.841	0.066
Number of metastatic lymph nodes in zone II (n)	0(0, 1.0)	0(0, 1.0)	0.363	0.717
Detection rate of lymph nodes in zone II (%)	0(0, 0.1)	0(0, 0.2)	0.769	0.442
Hospitalization cost (million yuan)	4.3±0.5	3.7±0.4	6.157	0.001

A statistically significant difference was observed in the incidence of postoperative complications between the two groups, which was lower in the endoscopic surgery group than that in the open surgery group (*p<*0.05).Table-II. Besides, hoarseness and hypoparathyroidism occurred temporarily after the operation.

**Table II:** Comparison of postoperative complications between the two groups.

	Endoscopic surgery group (n=34)	Open surgery group (n=52)	χ^2^	p
Hoarseness (n%)	5(14.7)	8(15.4)	0.007	0.932
Hypoparathyroidism (n%)	6(17.6)	11(21.2)	0.159	0.690
Hypocalcemia (n%)	5(14.7)	9(17.3)	0.102	0.749
Postoperative hematoma (n%)	0	6(11.5)	4.217	0.040
Total (n%)	10(29.4)	27(51.9)	4.250	0.039

There was no significant difference in postoperative neck pain scores between the two groups (*p>*0.05). While the postoperative neck numbness discomfort score, and satisfaction score with postoperative cosmetic effect in the endoscopic surgery group were better than those in the open surgery group, and the difference was statistically significant (*p<*0.05). Corresponding results are shown in Table-III.

**Table III:** Comparison of scores between the two groups

	Endoscopic surgery group (n=34)	Open surgery group (n=52)	Z	p
Neck pain score (point)	2(1, 2)	2(1, 2)	0.472	0.637
Neck numbness discomfort score (point)	1(1, 2)	2(1, 2)	1.987	0.047
Satisfaction score with postoperative cosmetic effect (point)	2(1, 2)	3(2, 3)	5.036	0.001

## DISCUSSION

PTC has a high risk of lymph node metastasis, with an incidence of about 30% ~ 80%.^15^ Traditional open surgery is a safe and effective choice for the treatment of lateral neck lymph node metastasis, while the formed scars after operation may disturb the normal daily life of patients.^16^ With the progress of the concept of biology-medicine-society, there is an extensive application of minimally invasive endoscopic surgery in clinic. Minimally invasive endoscopic cleaning of PTC refers to the process of establishing operation space through hook suspension technology via minimal neck incision, and completing the operation with the use of a 5 mm endoscope and ultrasonic knife.^17^ It has no visual dead angle during operation in the neck, and has significant advantages in the surgery of thyroid cancer. According to prior research,^18^ despite no significant difference between endoscopic surgery and traditional open surgery in efficacy and safety, the former surgery can significantly reduce the length of neck incision and has a cosmetic effect to some extent; and its cosmetic effect is superior to the latter one considering a lower position of the incision that is easy to cover. However, endoscopic surgery is a novel surgical approach that has not been fully popularized; and there are few physicians at home and abroad who acquire proficiency in applying this technique for the treatment of thyroid cancer, resulting in controversy in its safety for treating tumors.^19^

As discovered in our study, there was no significant difference in the number of dissected lymph nodes, the number of metastatic lymph nodes and the detection rate of lymph nodes in zone II between the two groups (*p>*0.05). It is suggested that endoscopic dissection has a similar therapeutic effect with traditional open surgery, both of which can play a satisfied role in lymph node dissection. In the aspect of safety, although there was no obvious difference in postoperative neck pain score between groups (*p>*0.05), the incidence of postoperative complications in the endoscopic surgery group was lower than that in the open surgery group (29.4% vs. 51.9%), accompanied by better postoperative neck numbness discomfort score and satisfaction score with postoperative cosmetic effect than those in the open group (*p<*0.05). These results support that endoscopic surgery is superior to open surgery in safety and can reduce the discomfort caused by surgery. In terms of potential causes, the use of endoscope intraoperatively can broaden the operation field of vision to accelerate the discovery of micro-nerves and blood vessels, resulting in the reduced risk of nerve and blood vessel injury during the operation;^20^ secondly, the application of endoscope offers flexible lighting that can change in intensity and lens distance, which may contribute to observing the tissue condition;^21^ and thirdly, endoscopic surgery has a small incision, which can meet the patients’ requirements for cosmetic effect; simultaneously, rather than traditional ligation, electrocoagulation is used to stop bleeding, which can reduce intraoperative blood loss and postoperative hematoma infection risk.

However and importantly, endoscopic surgery still has some defects, such as longer operation time and high hospitalization cost. As for the prolonged operative duration, the majority of physicians are unfamiliar with relevant operations considering a delayed popularization and application of endoscopic surgery in China. In this regard, the operation time of this surgery is relatively prolonged compared with traditional open surgery which has been widely and skillfully operated by doctors. While it has been reported that the theoretical operation time of endoscopic surgery can be shortened after the mastering of this technique^22^ since endoscopic surgery indeed has significant advantages in the operation processes with great difficulty and high precision requirements.

### The limitations of the study

It has the disadvantages of longer operation time and high hospitalization costs. Findings in our study require further verification based on larger sample size and longer duration of study owing to the smaller sample size and shorter follow-up period in the present study.

## CONCLUSIONS

Endoscopic-assisted lateral neck dissection can reduce intraoperative blood loss and postoperative complication incidence in the treatment of lateral neck lymph node metastasis of PTC.

### Authors’ Contributions:

**TM & SZ:** Designed this study, prepared this manuscript, are responsible and accountable for the accuracy and integrity of the work.

**DH & GZ:** Collected and analyzed clinical data.

**BC & NZ:** Data analysis, Significantly revised this manuscript.
